# First evidence of frugivory in *Myotis* (Chiroptera, Vespertilionidae, Myotinae)

**DOI:** 10.3897/BDJ.3.e6841

**Published:** 2015-11-09

**Authors:** Roberto Leonan Morim Novaes, Renan de França Souza, Edvandro Abreu Ribeiro, André Costa Siqueira, Alexandre Verçosa Greco, Ricardo Moratelli

**Affiliations:** ‡ Fundação Oswaldo Cruz, Rio de Janeiro, Brazil; § Universidade do Estado do Rio de Janeiro, Rio de Janeiro, Brazil; | Museu Nacional, Universidade Federal do Rio de Janeiro, Rio de Janeiro, Brazil

**Keywords:** Black Myotis, Brazilian Atlantic Forest, feeding habits, Neotropics

## Abstract

**Background:**

*Myotis* occurs from tropical to temperate regions throughout the globe, and it is the largest bat genus with more than 100 species. Most species are insect-eaters, but a few also feed on other invertebrates and fishes; there is no confirmed evidence of a plant item in their diet.

**New information:**

During fieldwork in the Brazilian Atlantic Forest, small seeds were retrieved from the feces of one adult female of the Black Myotis, *Myotis nigricans*—one of the most common Neotropical bats. In a germination experiment, 40% of those seeds grew into seedlings. Our findings are the first evidence of fruit consumption for any *Myotis* species. We reject a possible contamination because the cotton bag was never used before for bats. This study is the first evidence of frugivory in the genus *Myotis*.

## Introduction

Bats stand out among mammals in their remarkable variety of food habits (Wilson 1973). Considering the range of items included in their diet, we can split bats into those that feed on animals and those that feed on plants. Animal feeders comprise insect-eaters mostly, but several species are specialized in feeding on other invertebrates and terrestrial vertebrates, with a few species feeding on fishes and blood (Fenton 1992). Among those that feed on plants, there is an array of adaptations for feeding on fruits and flowers, with some species also feeding on leaves (Wilson 1973). This variety of habits has been arranged into seven feeding categories: fruit eaters, flower feeders, aerial insectivores, foliage gleaners, carnivores, fish eaters, and blood feeders (Wilson 1973); and a few species are classified as omnivores for feeding on insects, vertebrates, fruits, and flowers in a regular or seasonal way (Wilson 1973). Except for Phyllostomidae—which has representatives classified into quite distinct categories (as foliage gleaners, blood feeders, and fruit eaters)—most families comprise representatives of one or two related categories. But few species of animal feeders (including aerial insectivores and foliage gleaners) are known for including plant items in their diet, whereas some plant feeders may consume insects to complement their diets (Wilson 1973, Gardner 1977, Felix et al. 2013). These species are not considered omnivorous due to the low and sporadic representation of these different items in their diets.

*Myotis* (Vespertilionidae) is distributed worldwide and is the largest bat genus with more than 110 species (Simmons 2005, Ruedi et al. 2013). It has been recovered as monophyletic, and placed in its own subfamily—Myotinae (Hoofer and Van Den Bussche 2003). The genus is composed mainly of aerial insectivores, with a few foliage gleaners (that also feed on other invertebrates) and fish eaters (Wilson 1973). There is no confirmed evidence of plant items in the diet of a Myotinae bat. The Black Myotis, *Myotis nigricans* (Schinz, 1821), is among the most widely distributed mammals in the Neotropics, occurring from Mexico south into northern Argentina (LaVal 1973, Moratelli et al. 2013). It also can be considered one of the most common insectivorous bat species in South America, with records that range from well-preserved areas to habitats with different levels of human disturbance and encroachment, including rural and urban environments (Bianconi and Pedro 2007). Its biology and natural history have been extensively investigated (Wilson and Findley 1970, Wilson and Findley 1971, Wilson 1971i, LaVal 1973, Wilson and LaVal 1974, Siemers et al. 2001). The species has been classified as an aerial insectivorous that forages in forested and open habitats (Findley 1993), feeding on a variety of insects in the orders Coleoptera, Diptera, Ephemeroptera, Homoptera, Hymenoptera, and Lepidoptera, and small spiders as well (Wilson and LaVal 1974, Aguiar and Antonini 2008).

During fieldwork in the Atlantic Forest of Southeastern Brazil, small seeds were retrieved from the feces of one individual of *M. nigricans*. After using those seeds in a germination experiment, we report here the first evidence of fruit consumption for *M. nigricans* (and for the whole subfamily), and its possible role as a seed disperser.

## Material and Methods

The fieldwork was conducted in the Reserva Ecológica de Guapiaçu (REGUA), a 5,500 ha remnant of Atlantic Forest in the state of Rio de Janeiro. This protected area is connected with other conservation units that together comprise 60,000 ha of forests, comprising one of the largest Atlantic Forest remnants in the country. Fieldwork procedures followed the guidelines approved by the American Society of Mammalogists (Sikes et al. 2011). Animals captured were removed from the nets, and kept alone in silk bags before handling for identification and biometry. To study the diet of this local assemblage, at the end of each capture section cotton bags were examined for feces with food items potentially identifiable. Feces were washed, and the seeds found were separated into morphotypes. From the set of each morphotype obtained from each animal, two seeds were preserved in ethanol 70° GL, and the rest was used in germination experiments. These experiments were performed in Petri dishes with moistened filter paper, under natural conditions of light and humidity.

## Results and Discussion

The individual of *M. nigricans* is an adult female, captured on 6 January 2012, in a ground-level mist-net (9 m long, 3 m high, 20 mm mesh-size) setup in a continuous forest edge (22°25'19.77" S, 42°44'58.84" W, elevation ca. 90 m). The animal was removed from the net, and kept alone in a cotton bag for about 30 minutes before handling. It was collected and deposited as a voucher in the Museu Nacional (MN 79898), Rio de Janeiro. The analysis of the feces in the cotton bag revealed 12 seeds of an unidentified plant, each measuring about 1 mm in diameter. Two of them were preserved, and the remaining (*N* = 10) were used in the germination experiment. From the 10 seeds used, 40% germinated and grew into seedlings after twelve days.

The presence of seeds in the feces of an individual of *M. nigricans* is the first evidence of a plant item in the diet of a *Myotis* species. This finding is new for *M. nigricans* and for the whole genus (and subfamily), but need further and careful investigation. We reject possible contamination (feces of other animal in the cotton bag) because we were using those cotton bags for the first time, and that was the first capture section. Also, cotton bags were cleaned, and animals were kept alone in the bag. We also reject possible accidental fruit consumption during predation because *M. nigricans* is an aerial insectivore, catching insects during flight.

Although the use of plant items in the diet of *M. nigricans* was totally unexpected, plant remains in the stomach of one specimen from Costa Rica was once reported (Alberto Cadena in personal communication to Wilson and LaVal 1974). Several species are known for complementing their diets with items other than those that have mainly influenced their morphology (Ferrarezi and Gimenez 1996). Also, other bat species previously considered strictly insectivorous have been reported to consume plant items in their diets. As examples, Frick et al. (2009) described the facultative nectarivory for *Antrozous pallidus* (Vespertilionidae) in the United States; and Gonçalves et al. (2007) reported *Noctilio albiventris* (Noctilionidae) feeding on fruits, nectar and pollen in the Brazilian wetlands of Pantanal. Depending on local environmental constrains, these plant items can be important nutritional complements for the diets of insectivorous species, and bats can consume them sporadically or seasonally, depending on the availability of their favored food sources (Wolf et al. 2002, Frick et al. 2009, Felix et al. 2013). The discovery of a well-studied species, previously considered strictly insectivorous, foraging on fruits shows how little we know about the biology and natural history of Neotropical bats.
